# Structural basis for inhibition of the type I-F CRISPR–Cas surveillance complex by AcrIF4, AcrIF7 and AcrIF14

**DOI:** 10.1093/nar/gkaa1199

**Published:** 2020-12-17

**Authors:** Clinton Gabel, Zhuang Li, Heng Zhang, Leifu Chang

**Affiliations:** Department of Biological Sciences, Purdue University, 915 W. State Street, West Lafayette, IN 47907, USA; Department of Biological Sciences, Purdue University, 915 W. State Street, West Lafayette, IN 47907, USA; Department of Biological Sciences, Purdue University, 915 W. State Street, West Lafayette, IN 47907, USA; Department of Biological Sciences, Purdue University, 915 W. State Street, West Lafayette, IN 47907, USA; Purdue University Center for Cancer Research, Purdue University, 915 W. State Street, West Lafayette, IN 47907, USA

## Abstract

CRISPR–Cas systems are adaptive immune systems in bacteria and archaea to defend against mobile genetic elements (MGEs) and have been repurposed as genome editing tools. Anti-CRISPR (Acr) proteins are produced by MGEs to counteract CRISPR–Cas systems and can be used to regulate genome editing by CRISPR techniques. Here, we report the cryo-EM structures of three type I-F Acr proteins, AcrIF4, AcrIF7 and AcrIF14, bound to the type I-F CRISPR–Cas surveillance complex (the Csy complex) from *Pseudomonas aeruginosa*. AcrIF4 binds to an unprecedented site on the C-terminal helical bundle of Cas8f subunit, precluding conformational changes required for activation of the Csy complex. AcrIF7 mimics the PAM duplex of target DNA and is bound to the N-terminal DNA vise of Cas8f. Two copies of AcrIF14 bind to the thumb domains of Cas7.4f and Cas7.6f, preventing hybridization between target DNA and the crRNA. Our results reveal structural detail of three AcrIF proteins, each binding to a different site on the Csy complex for inhibiting degradation of MGEs.

## INTRODUCTION

To survive under the constant pressure of phage infection, bacteria have developed not only diverse innate immune strategies, but also adaptive immune systems known as the Clustered Regularly Interspaced Palindromic Repeats (CRISPR) and CRISPR-associated protein (Cas) systems (1,2). These CRISPR–Cas systems fight phage and other mobile genetic elements (MGEs) through a three-stage process: adaptation, expression and interference. During the adaptation stage, fragments of viral DNAs (protospacers) are processed and integrated into the CRISPR array as spacers. In the expression stage, a precursor transcript (pre-crRNA) is transcribed and matured into small CRISPR RNA (crRNA) by Cas proteins or RNase III. In the interference stage, crRNA-guided effector nucleases cleave and degrade the invasive nucleic acids with a protospacer (1,2). The effector nucleases recognize 2–6 bp protospacer adjacent motifs (PAMs) of virus DNA which is absent in the bacterial host's own DNA to avoid self-targeting of the CRISPR array. Interactions between bacteria and phage have resulted in extreme diversification of CRISPR–Cas systems, which are grouped into two distinct classes comprising six types (I–VI) based on CRISPR locus organization and the Cas gene composition (3,4). The class 1 system (types I, III and IV) employs multi-protein effector complexes to cleave foreign nucleic acids, while the class 2 system (types II, V and VI) utilizes a single multi-domain Cas effector. In addition to acting as bacterial immune systems, these CRISPR–Cas systems have been repurposed for genome editing and molecular diagnostic applications that are transforming biomedical research (5–7).

However, phages have developed counter-adaptions to bacterial anti-phage strategies, instigating a molecular arms race between phage and bacteria (8). For instance, in response to the CRISPR–Cas systems, phages have evolved anti-CRISPR (Acr) proteins to evade CRISPR interference (9–11), often by blocking one of three stages of CRISPR–Cas action. Acr proteins are small proteins with no common sequence or structural motifs. To date, diverse Acr proteins have been discovered through functional screening and bioinformatic analysis (12,13)

The first Acr proteins (AcrIF1–5) were discovered in 2013 as genes that inactivated the type I-F CRISPR–Cas surveillance complex (the Csy complex) in *Pseudomonas aeruginosa* (14) found from a diverse array of *P. aeruginosa* phages including JBD30, D3112, JDB5, JBD26 and JBD5 for AcrIF1–5, respectively. Since then, nine additional AcrIF families (AcrIF6–14) were reported (14–16). Recently, more AcrIF inhibitors have been found that extend to AcrIF24 (17). The Csy complex is composed of four Cas proteins including Cas5f, Cas6f, Cas7f and Cas8f in a stoichiometry of 1:1:6:1, and a 60-nt crRNA that integrates all these protein subunits (18–22). Target DNA binding and R-loop formation induce conformational changes in the Csy complex, enabling it to recruit the Cas2/3 nuclease for degradation of the invading MGEs (21). A common inhibition strategy of Acr proteins is blocking target DNA binding. Such mechanisms are exemplified by structural studies of AcrIF proteins including AcrIF1 (18–20), AcrIF2 (18–20), AcrIF6 (23), AcrIF8 (23), AcrIF9 (22,23) and AcrIF10 (19). AcrIF3 adopts a similar structure to the C-terminal helical bundle of Cas8f, competitively binding to the Cas2/3 nuclease and preventing its recruitment to the Csy complex (21,24).

In this study, we elucidate the structures of AcrIF4, AcrIF7 and AcrIF14 bound to the Csy complex. These structures provide insights into the mechanisms by which AcrIF proteins interact with different sites on the Csy complex to suppress different steps of the interference stage of type I-F CRISPR–Cas systems.

## MATERIALS AND METHODS

### Plasmid constructs and protein purification

DNA sequences of AcrIF4, AcrIF7 and AcrIF14 were ordered as gBlocks from Integrated DNA Technologies, Inc., which were cloned individually into pET His6 Sumo TEV LIC cloning vectors (1S) (Addgene # 29659) using the Gibson Assembly® Master Mix (NEB, Cat. # E2611S). After sequence verification, these plasmids were transformed into BL21 (DE3) cells for expression in Terrific Broth medium. Protein expression was induced by 0.5 mM isopropyl β-d-1-thiogalactopyranoside (IPTG) at 16°C overnight. Cell pellets were resuspended in lysis buffer containing 50 mM HEPES (pH 7.5), 500 mM NaCl, 5% glycerol, 5 mM β-meracptoethanol, 0.2 mM phenylmethylsulfonyl fluoride (PMSF), and disrupted by sonication. After centrifugation, the supernatant was loaded in a HisTrap HP column. AcrF proteins were eluted with a stepwise gradient of 1.0 M imidazole, digested with TEV protease overnight for removal of 6XHis and SUMO-tags, and purified with ion-exchange chromatography using either a Heparin HiTrap Q HP or Heparin HiTrap SP HP depending on protein isoelectric point (p*I*). AcrIF proteins were then concentrated and further purified over a Superdex 200 column (GE Healthcare) in a buffer containing 20 mM HEPES (pH 7.5), 150 mM NaCl, 5% glycerol and 1 mM Tris(2-carboxyethyl)phosphine hydrochloride (TCEP).

For purification of the PA14 Csy complex, the pCsy_complex plasmid (Addgene ID# 89232) and pCRISPR_DMS3g24 (Addgene ID # 89244) were co-transformed into BL21 (DE3) cells for expression. Cell pellets were resuspended in lysis buffer containing 50 mM HEPES (pH 7.5), 300 mM KCl, 5% glycerol, 0.2 mM PMSF, 1 mM TCEP and cOmplete™ protease inhibitor (Roche, 04693132001), and disrupted by sonication. Intact Csy complex was purified by a HisTrap HP column (GE Healthcare) as previously described for the AcrIF proteins, and the buffer was exchanged overnight into a buffer containing 50 mM HEPES (pH 7.5), 150 mM KCl, 5% glycerol, and 1 mM TCEP. After ion-exchange chromatography using a Heparin HiTrap Q column (GE Healthcare), the Csy complex was further purified using a Superdex 200 column (GE Healthcare) equilibrated in a buffer containing 20 mM HEPES (pH 7.5), 100 mM KCl, 5% glycerol, and 1 mM TCEP.

### Complex assembly

To assemble Csy-AcrIF complexes, purified Csy was incubated with AcrIF proteins at a molar ratio of 1:10 for 1 h on ice, and then subjected to size exclusion chromatography over a Superdex 200 column (GE Healthcare) equilibrated in buffer containing 20 mM HEPES (pH 7.5), 100 mM KCl, 5% glycerol and 1 mM TCEP, followed by SDS-PAGE analysis of the elution fractions.

To test whether AcrIF4, AcrIF7 and AcrIF14 can interact with the Csy complex simultaneously, Csy, AcrIF4, AcrIF7 and AcrIF14 were incubated at molar ratio of 1:3:3:3 for 1 h on ice, and then subjected to size exclusion chromatography over a Superdex 200 column (GE Healthcare) equilibrated in buffer containing 50 mM HEPES (pH 7.5), 100 mM KCl, 5% glycerol and 1 mM TCEP, followed by SDS-PAGE analysis of the elution fractions.

### Electromobility Shift Assays (EMSA)

A dsDNA Substrate was prepared by mixing two complementary ssDNAs purchased from IDT. The target (5′-CAGGTAGACGCGGACATCAAGCCCGCCGTGAACAGGTAGACGCGGACATCAAGCCCGCCGTGAACAGGTAGACGCGGACATCAAGCCCG-3) and non-target strands (3′-GTCCATCTGCGCCTGTAGTTCGGGCGGCACTTGTCCATCTGCGCCTGTAGTTCGGGCGGCACTTGTCCATCTGCGCCTGTAGTTCGGGC-5′) were mixed and then denatured at 95°C for 5 minutes and then allowed to cool to room temperature before use in binding assays.

Binding assays were performed with 400 nM Csy and increasing concentrations of AcrIF4 or AcrIF7 (0.4, 4.0, 8.0, 16, 40 and 80 μM) in reaction buffer (20 mM HEPES, pH 7.5, 150 mM KCl, 1 mM TCEP, 5% glycerol, 2 mM MgCl_2_). Csy and inhibitors were first incubated for 1 h on ice before addition of dsDNA to a final concentration of 100 nM. Samples were then heated to 37°C for 30 min with mild agitation. Samples were then removed from heat, and reaction products were run on native 6% polyacrylamide TBE gels (DNA retardation gels, ThermoFisher). Gels were stained with SYBR Green Nucleic Acid Stain (ThermoFisher) and imaged with a GE Healthcare ImageQuant LAS 4000.

### Electron microscopy

Aliquots of 3 μl Csy-AcrIF4, Csy-AcrIF7 and Csy-AcrIF14 at 0.5 mg/ml were applied to glow-discharged UltrAuFoil holey gold grids (R1.2/1.3, 300 mesh). The grids were blotted for 2.5 s and plunged into liquid ethane using a ThermoFisher Scientific Mark IV Vitrobot. Cryo-EM data were collected with a Titan Krios microscope (FEI) operated at 300 kV and images were collected using Leginon (25) at a nominal magnification of 81,000x (resulting in a calibrated physical pixel size of 1.05 Å/pixel) with a defocus range of 1.2–2.5 μm. The images were recorded on a K3 electron direct detector in super-resolution mode at the end of a GIF-Quantum energy filter operated with a slit width of 20 eV. A dose rate of 20 electrons per pixel per second and an exposure time of 3.12 s were used, generating 40 movie frames with a total dose of ∼54 electrons/Å^2^. Statistics for cryo-EM data are listed in Table 1.

**Table 1.** Cryo-EM data collection, refinement and validation statistics

	Csy-AcrIF4 (EMD-22582, PDB 7JZW)	Csy-AcrIF7 (EMD-22583, PDB 7JZX)	Csy-AcrIF14 (EMD-22585, PDB 7JZZ)
**Data collection and processing**			
Magnification	81 000	81 000	81 000
Voltage (kV)	300	300	300
Electron exposure (e^–^/Å^2^)	54	54	54
Defocus range (μm)	1.5–2.5	1.5–2.5	1.5–2.5
Pixel size (Å)	1.05	1.05	1.05
Symmetry imposed	*C1*	*C1*	*C1*
Initial particle images (no.)	1 765,062	1 627 883	1 217 862
Final particle images (no.)	766 782	502 177	226 089
Map resolution (Å)	3.2	3.4	3.2
FSC threshold	0.143	0.143	0.143
Map resolution range (Å)	3–4.2	3.2–4.4	3–4.2
**Refinement**			
Initial model used	PDB 6NE0	PDB 7JZW	PDB 7JZW
Model resolution (Å)	3.2	3.4	3.2
FSC threshold	0.5	0.5	0.5
Model resolution range (Å)	3.2–50	3.4–50	3.2–50
Map sharpening *B* factor (Å^2^)	–112	–129	–86
Model composition			
Non-hydrogen atoms	24 266	23 948	24 767
Protein residues	2986	2954	3092
Nucleotides	61	61	61
Ligands	0	0	0
*B* factors (Å^2^)			
Protein	28.49	65.26	48.40
Nucleotide	58.01	75.10	81.07
R.m.s. deviations			
Bond lengths (Å)	0.010	0.009	0.010
Bond angles (°)	0.977	0.921	0.980
**Validation**			
MolProbity score	1.75	1.73	1.76
Clashscore	5.65	5.33	5.72
Poor rotamers (%)	0.13	0.40	0.00
Ramachandran plot			
Favored (%)	93.00	92.99	93.07
Allowed (%)	7.00	7.01	6.93
Disallowed (%)	0.00	0.00	0.00

### Image processing

The movie frames were imported to RELION-3 (26). Movie frames were aligned using MotionCor2 (27) with a binning factor of 2. Contrast transfer function (CTF) parameters were estimated using Gctf (28). A few thousand particles were auto-picked without template to generate 2D averages for subsequent template-based auto-picking. The auto-picked and extracted particle dataset were split into batches for 2D classifications, which were used to exclude false and bad particles that fall into 2D averages with poor features. Particles from different views were used to generate an initial model in cryoSPARC (29). 3D classification was then performed, followed by 3D refinement using particles in good 3D classes. Focused refinements around the inhibitors were further performed to improve the local resolutions.

For the Csy-AcrIF4 dataset, 1 765 062 particles were auto-picked and extracted from the dose weighted micrographs. 1 123 229 particles were selected from 2D classification and used for 3D classification. 766 782 particles were selected from 3D classification and used for final 3D refinement. For the Csy-AcrIF7 dataset, 1 627 883 particles were auto-picked and extracted from the dose weighted micrographs. 989 921 particles were selected from 2D classification and used for 3D classification. 502 177 particles were selected from 3D classification and used for final 3D refinement. For the Csy-AcrIF14 dataset, 1 217 862 particles were auto-picked and extracted from the dose weighted micrographs. 1 174 148 particles were selected from 2D classification and used for 3D classification. 226 089 particles were selected from 3D classification and used for final 3D refinement. Statistics of cryo-EM image processing are summarized in Table 1.

### Model building and refinement

*De novo* model building of AcrIF4, AcrIF7 and AcrIF14 structures were performed manually in COOT (30). Secondary structure predictions by PSIPRED (31) were used to assist manual building. To build models of the Csy complex, each subunit of the structure of the Csy-target DNA complex (PDB:6NE0) was fitted into the maps as a rigid-body in UCSF Chimera (32) and manually adjusted in COOT. Refinement of the structure models against corresponding maps were performed using *phenix.real_space_refine* tool in Phenix (33).

### Visualization

Figures were generated using PyMOL and UCSF Chimera (32).

## RESULTS

### Structural determination of Csy-AcrIF complexes

**Figure 1.**
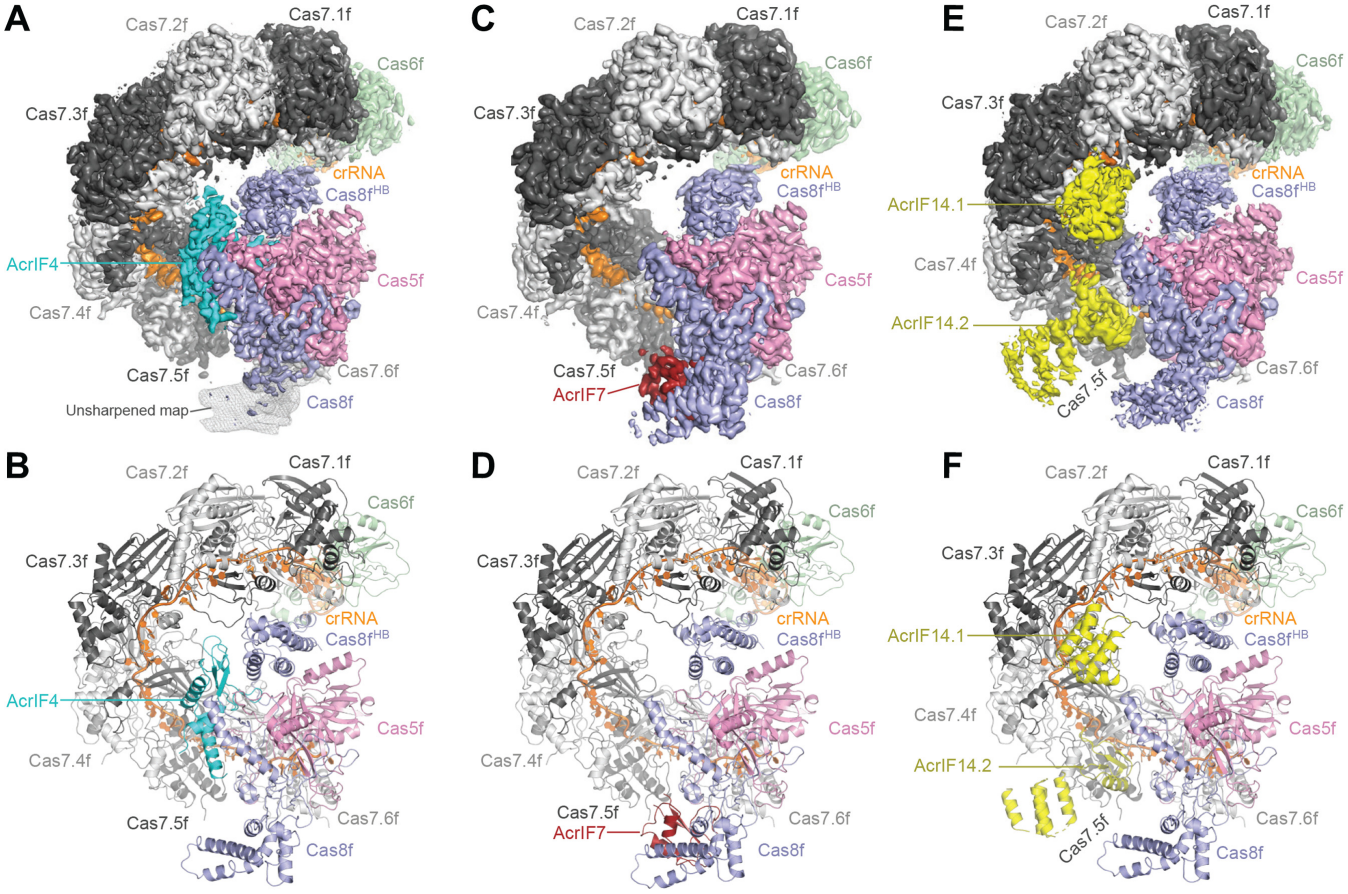
Overall structures of the Csy-AcrIF4, Csy-AcrIF7 and Csy-AcrIF14 complexes. (**A**) Cryo-EM map of Csy-AcrIF4 in surface representation with each subunit color-coded. AcrIF4 is in cyan. The unsharpened map of the DNA vise in Cas8f is shown in mesh. (**B**) Atomic model of Csy-AcrIF4 in cartoon representation with each subunit color-coded as in A. (**C**) Cryo-EM map of Csy-AcrIF7 with each subunit color-coded. AcrIF7 is in firebrick red. (**D**) Atomic model of Csy-AcrIF7 in cartoon representation with each subunit color-coded as in C. (**E**) Cryo-EM map of Csy-AcrIF14 with each subunit color-coded. Two copies of AcrIF14 are in yellow. (**F**) Atomic model of Csy-AcrIF14 in cartoon representation with each subunit color-coded as in E.

Using single-particle cryo-EM, we determined the structures of the Csy complex in *Pseudomonas aeruginosa* bound to inhibitors AcrIF4, AcrIF7, and AcrIF14 at resolutions of 3.2, 3.4 and 3.2 Å, respectively (Figure 1 and Supplementary Figures S1–S4, and Table 1). The cryo-EM maps were sufficient for building the atomic models of individual AcrIF proteins *de novo* and rebuilding of the components in the Csy complex.

Consistent with previously reported structures, the Csy complex adopts a helically twisted ‘G’ shape with Cas5f, Cas6f, Cas7f and Cas8f protein components integrated by the crRNA (Figure 1 and Supplementary Figure S5). Cas6f is located at the 3′ hairpin of the crRNA distal to the PAM binding site, forming the head of the complex, whereas Cas8f and Cas5f are located at the 5′ handle of the crRNA forming the tail of the complex (PAM-proximal end). Between the head and the tail are six interlocking Cas7f molecules (Cas7.1f-Cas7.6f), forming the spiral backbone of the structure (18–20). All three AcrIF-bound Csy structures share a similar conformation to the Csy complex alone, indicating that AcrIF binding does not induce significant conformational changes, in contrast to the conformational changes observed after target DNA binding (21).

### Structure of Csy-AcrIF4

**Figure 2.**
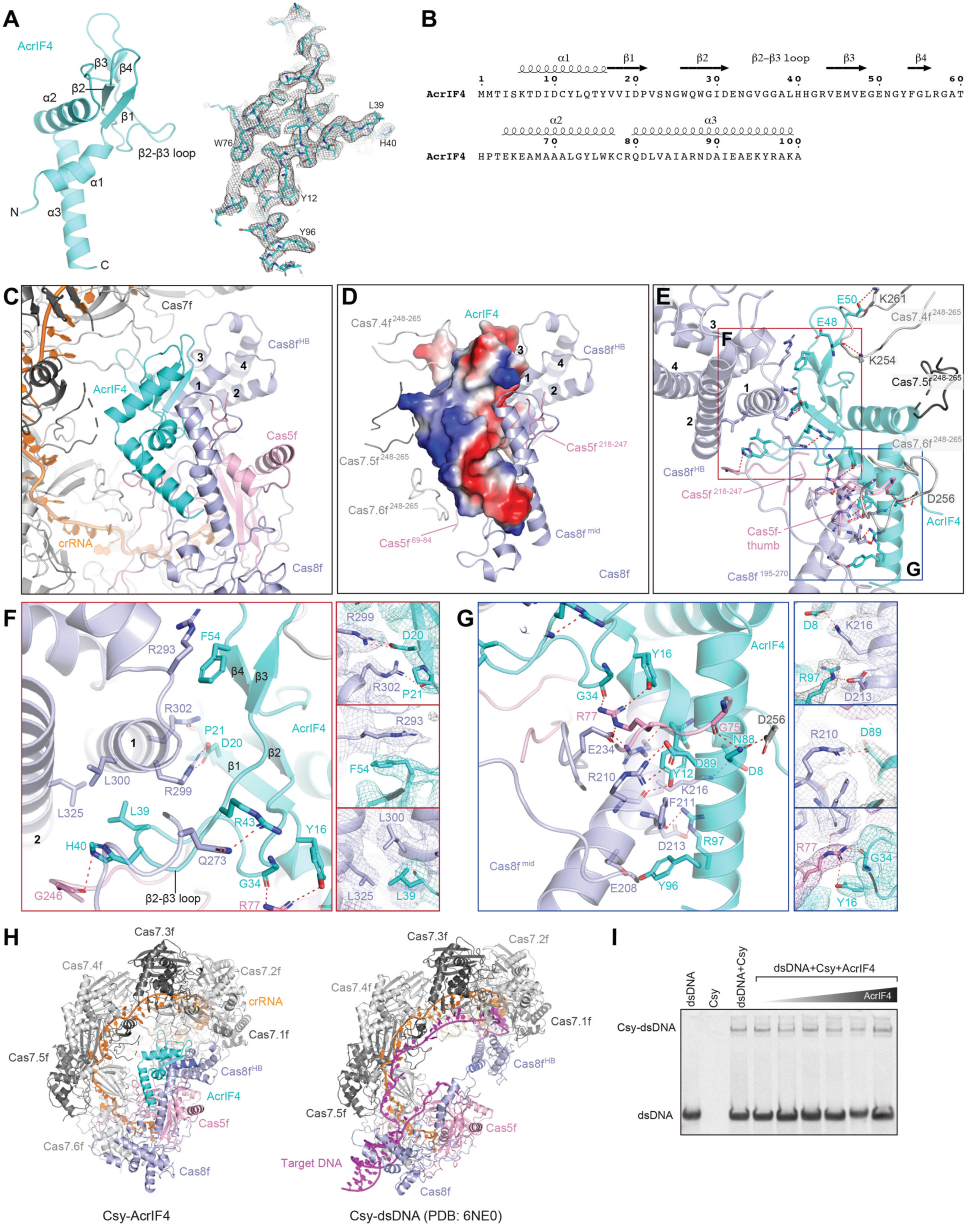
Structure of AcrIF4 and its interactions with the Csy complex. (**A**) Atomic model of AcrIF4 in cartoon representation. Stick representation of AcrIF4 with corresponding cryo-EM map in mesh is shown on the right. (**B**) Amino acid sequence of AcrIF4 with secondary structures labeled. (**C**) Focused view of AcrIF4 and surrounding components in the Csy–AcrIF4 complex. (**D**) Surface potential of AcrIF4 shown in the same view as in C. The subunits of Csy in close contact with AcrIF4 are shown as cartoon representation, including Cas8f^mid^, Cas8f^HB^, loops in Cas7.4f (Cas7.4f^248–265^), Cas7.5f (Cas7.5f^248–265^), Cas7.6f (Cas7.6^248–265^) and Cas5f (Cas5f^69–84^and Cas5f^218–247^). (**E**) Detailed interactions between AcrIF4 and the Csy complex. Interactions are indicated by red dashed lines. Regions indicated by red and blue squares are shown in enlarged view in panels F and G, respectively. (**F**) Detailed interactions between the β-strand domain of AcrIF4 and Cas8f^HB^ of the Csy complex. Focused views of side chain interactions are shown on the right, with cryo-EM density map shown in mesh. (**G**) Detailed interactions between the helical domain of AcrIF4 and Cas8f^mid^ of Csy. Focused views of side chain interactions are shown on the right, with cryo-EM density map shown in mesh. (**H**) Side-by-side comparison of the Csy-AcrIF4 and Csy-dsDNA (PDB:6NE0) structures. (**I**) EMSA assays of dsDNA (100 nM) binding by the Csy complex (400 nM) in the presence of different concentrations of AcrIF4 (from left to right: 0.4, 4.0, 8.0, 16.0, 40.0 and 80.0 μM).

AcrIF4 is a 100-residue protein from the JBD5 phage (14) composed of a helical domain and a β-strand domain. The helical domain contains α1 at the N-terminus and α2 and α3 at the C-terminus, while the β-strand domain contains two pairs of anti-parallel β strands (β1–β2 and β3–β4) with a 34-residue loop between β2 and β3 (a.a. 31–64) (Figure 2A, B). A Dali search (34) revealed that AcrIF4 shows no obvious resemblance to known protein structures in the PDB.

AcrIF4 is clamped between the spiral backbone of the Csy complex and Cas8f (Figures 1A, B and 2C). AcrIF4 primarily binds to Cas8f on its middle region (Cas8f^mid^, a.a. 195–272) and C-terminal four-helix bundle (Cas8f^HB^, a.a. 273–434) through a negatively charged surface, with additional contacts to Cas7.4f, Cas7.5f, Cas7.6f and Cas5f (Figure 2C–E). The β-strand domain of AcrIF4 fits against helix α1 of Cas8f^HB^. In the middle, D20 and P21 of AcrIF4 engage R299 and R302 of the helix α1 of Cas8f^HB^ by a salt bridge and a hydrogen bond, respectively (Figure 2F). On one side, F54 of AcrIF4 forms a cation-pi stacking interaction with R293 of Cas8f^HB^; on the other side, L39 of AcrIF4 forms hydrophobic interactions with L300 and L325 of Cas8f^HB^ (Figure 2F). The helical domain of AcrIF4 interacts with Cas8f^mid^ through a network of contacts including three salt bridges (AcrIF4-D8:Cas8f^mid^-K216, AcrIF4-D89:Cas8f^mid^-R210, and AcrIF4-R97:Cas8f-D213) and extensive hydrogen bonds (Figure 2G). The helical domain of AcrIF4 also makes contacts with a loop within Cas5f (a.a. 69–84). For example, G34 and Y16 of AcrIF4 both engage R77 of Cas5f (Figure 2G).

Target DNA binding to the Csy complex induces a 180° rotation of Cas8f^HB^ (Figure 2H and Movie S1), which is essential for recruitment of the Cas2/3 nuclease for cleavage of the substrate (21). These interactions observed between AcrIF4 and Cas8f^HB^ indicate that AcrIF4 may prevent the rotation of Cas8f^HB^, thereby locking the Csy complex in an inactive state (Figure 2F and Movie S1). Structural comparison between the Csy-AcrF4 and Csy-target dsDNA suggest that AcrF4 does not compete with PAM recognition or hybridization between crRNA and the target strand of dsDNA (Figure 2H). Consistent with this observation, electromobility shift assays (EMSAs) showed that the Csy complex was able to bind to dsDNA substrate in the presence of AcrIF4 (Figure 2I).

### Structure of Csy-AcrIF7

**Figure 3.**
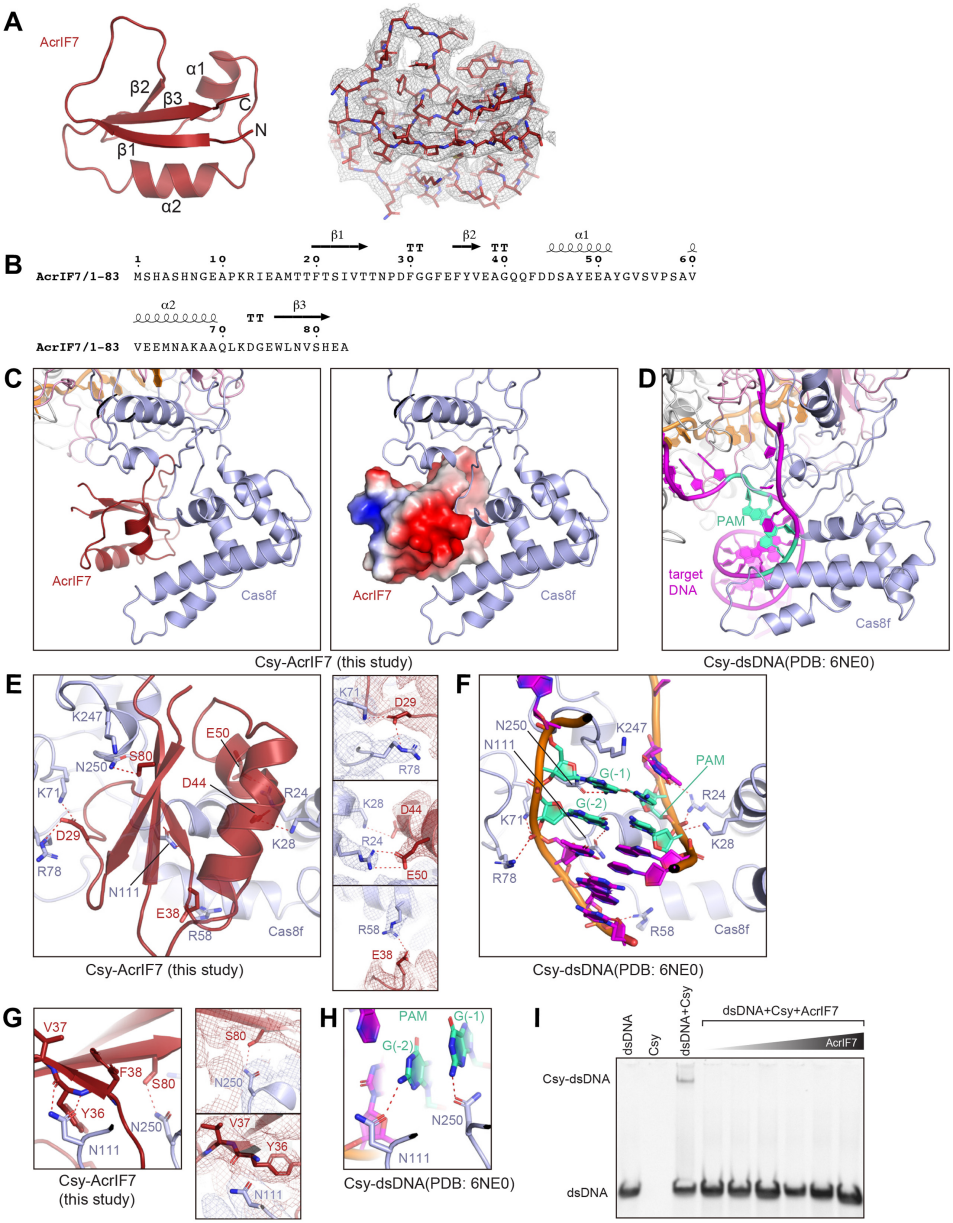
Structure of AcrIF7 and its interactions with the Csy complex. (**A**) Atomic model of AcrIF7 in cartoon representation. Stick representation of AcrIF7 with corresponding cryo-EM map in mesh is shown on the right. (**B**) Amino acid sequence of AcrIF7 with secondary structures labeled. (**C**) Focused view of AcrIF7 represented in cartoon (left) and surface potential (right) in the Csy-AcrIF7 complex. (**D**) Focused view of the PAM duplex of target DNA in the Csy-dsDNA complex (PDB: 6NE0). (**E**) Detailed interactions between AcrIF7 and the Csy complex. Interactions are indicated by red dashed lines. Focused views of side chain interactions are shown on the right, with cryo-EM density map in mesh. (**F**) Detailed interactions between the PAM duplex of target DNA and the Csy complex, in the same view as in E. Interactions are indicated by red dashed lines. (**G**) N111 and N250 of Cas8f (key residues involved in PAM recognition) directly interact with AcrIF7. Focused views of side chain interactions are shown on the right, with cryo-EM density map in mesh. (**H**) Interactions between N111 and N250 of Cas8f and the PAM sequence of target DNA, in the same view as in G. (**I**) EMSA assays of dsDNA (100 nM) binding by the Csy complex (400 nM) in the presence of different concentrations of AcrIF7 (from left to right: 0.4, 4.0, 8.0, 16.0, 40.0 and 80.0 μM).

The 83-residue AcrIF7 from a *P. aeruginosa* prophage (15) adopts a globular shape with an anti-parallel β-sheet core flanked by two helices in an β1β2α1α2β3 topology, consistent with a recently reported NMR structure of AcrIF7 (35) (Figure 3A, B).

AcrIF7 binds to the positively charged ‘DNA vise’ at the N-terminal domain of Cas8f (a.a. 1–195), where the PAM duplex of the target DNA binds (Figure 3C,D). Comparison of the structures of Csy-AcrIF7 and Csy-target DNA (PDB: 6NE0) shows that AcrIF7 competitively binds to the ‘DNA vise’ of Cas8f primarily through charged interactions. Specifically, D29 of AcrIF7 interacts with K71 and R78 of Cas8f (Figure 3E), mirroring the phosphate group of G(–2) of the target strand within the PAM sequence of target DNA (Figure 3F). D44 and E50 interact with K28 and R24, respectively (Figure 3E), mimicking the phosphate groups of C(–2) and C(–1) of the non-target strand of target DNA (Figure 3F). E38 interacts with R58, imitating another phosphate group at position –4 of the target strand. The interactions revealed by our structure are supported by previous mutagenesis analysis, which showed that mutations of D29 and E49/E50 to positively charged residues reduced the affinity of AcrIF7 to the Cas8f–Cas5f dimer by 50–100 fold (35).

In addition, S80 of AcrIF7 hydrogen bonds with N250 of Cas8f, while Y36 and V37 of AcrIF7 hydrogen bond with N111 of Cas8f (Figure 3G). N250 and N111 are reported to recognize the PAM duplex on the target DNA (Figure 3H). This result suggests that AcrIF7 not only mimics the surface potential of DNA substrate but also the bases in the PAM sequence, thereby blocking the initial step for target DNA recognition by the Csy complex. Consistent with this hypothesis, EMSAs showed that AcrIF7 effectively blocks target dsDNA binding to the Csy complex (Figure 3I).

### Structure of Csy-AcrIF14

**Figure 4.**
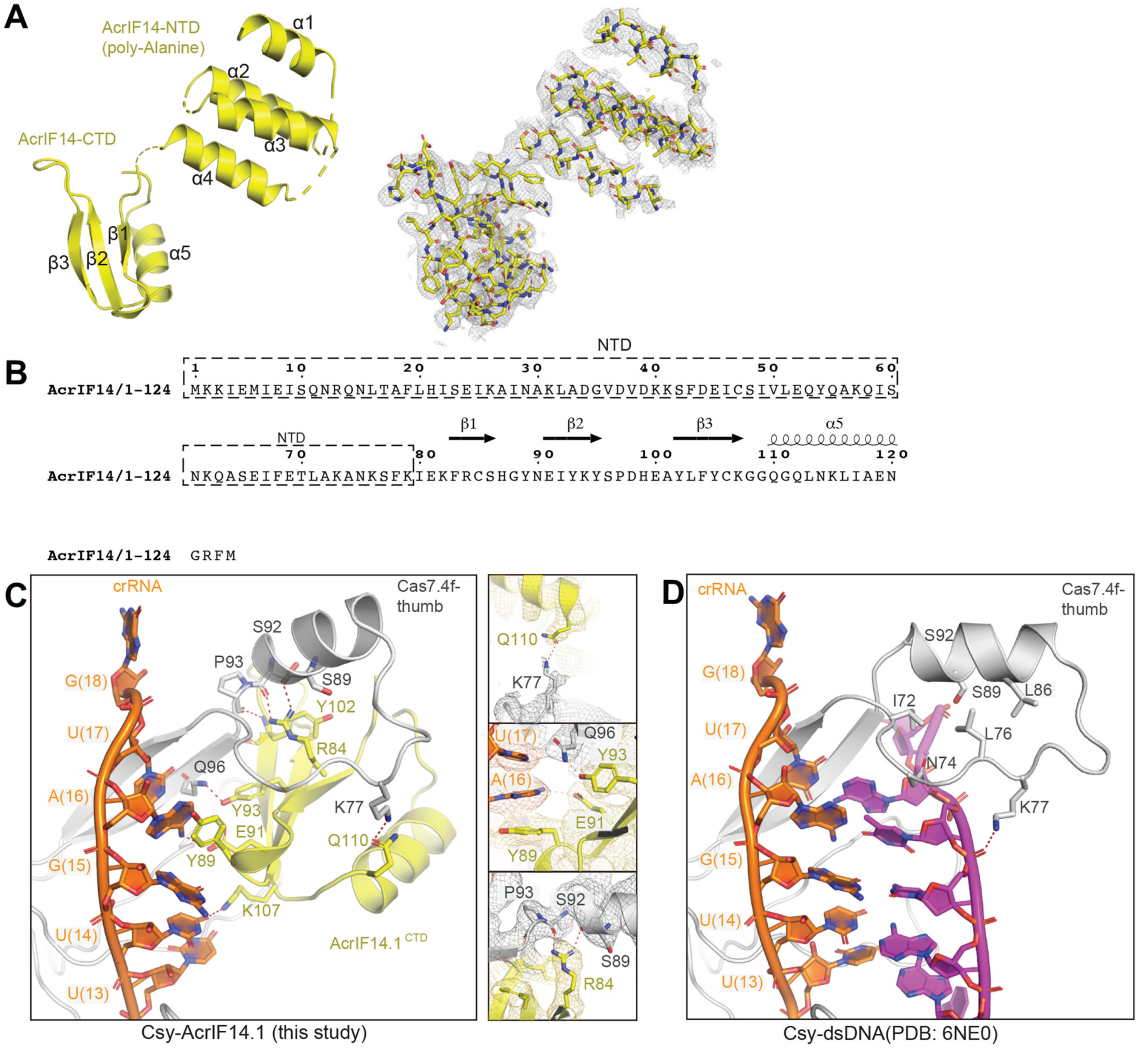
Structure of AcrIF14 and its interactions with the Csy complex. (**A**) Atomic model of AcrIF14 in cartoon representation. Stick representation of AcrIF14 with corresponding cryo-EM map in mesh is shown on the right. (**B**) Amino acid sequence of AcrIF14 with secondary structures labeled. The NTD in dashed box was built as a poly-alanine model. (**C**) Detailed interactions between AcrIF14.1 and Cas7.4f. Interactions are indicated by red dashed lines. Focused views of side chain interactions are shown on the right, with cryo-EM density map in mesh. (**D**) Detailed interactions between target DNA and Cas7.4f, in the same view as in C.

There are two AcrIF14 molecules bound to the Csy complex (Figure 1E,F). The 124-residue AcrIF14 from the Mcat5 phage (16) is a comparatively large AcrIF protein and is divided into two domains (Figure 4A,B). The C-terminal domain (CTD, aa 80–124) is composed of an anti-parallel β-sheet followed by a helix (α5). AcrIF14^CTD^ is well ordered in the Csy–AcrIF14 complex and the cryo-EM density allowed its atomic model building. In contrast, the N-terminal domain (NTD, aa 1–80) shows no direct contact with the Csy complex and is built as a poly-alanine model because of resolution limitations in this region. The NTD of AcrIF14 adopts a helix-turn-helix (HTH) fold. Fusion with the HTH and other domains has been observed in a few Acr proteins, including AcrIIA1 (36), AcrIIA13–15 (37), AcrVA4 (38–40). These domains may play a role in DNA binding and regulation of the function of Acr proteins.

Two copies of AcrIF14^CTD^ interact with the thumb domains of Cas7.4f and Cas7.6f, respectively, mainly through polar interactions. R84 of AcrIF14^CTD^ forms hydrogen bonds with the carbonyl groups of S89 and S92 of the thumb domains of Cas7f (Figure 4C). Q110 and Y93 of AcrIF14^CTD^ engages K77 and Q96 of Cas7f, respectively (Figure 4C). In addition to the thumb domains of Cas7.4f and Cas7.6f, AcrIF14^CTD^ also interacts with crRNA, reminiscent of AcrIF9 (22,23). Y89 of AcrIF14.1 packs against A(16) augmented by a potential hydrogen bond between E91 and A(16) (Figure 4C). Similarly, AcrF14.2 makes analogous contacts with the crRNA but with a different base, A(12) (Supplementary Figure S6A,B). The pi stacking interactions between Y89 of AcrF14 and the crRNA are sequence independent and will likely be maintained with different crRNAs. A comparison between the Csy-AcrF14 structure and the Csy-dsDNA structure suggests that AcrIF14^CTD^ competes with target DNA to bind the crRNA and the thumb domains of Cas7.4f and Cas7.6f (Figure 4C, D). Simulation of AcrIF14 binding to other Cas7f subunits shows severe steric clashes, explaining why AcrIF14 selectively binds to Cas7.4f and Cas7.6f (Supplementary Figure S6C-F). Interestingly, AcrIF7 and AcrIF14 can interact with Csy simultaneously (Supplementary Figure S1D), reminiscent to AcrIF2 and AcrIF1 which were shown to bind Csy simultaneously (18,20).

## DISCUSSION

To direct target DNA degradation by the Csy complex, at least three major sequential steps are required. First, the PAM duplex of the target DNA is recognized by the ‘DNA vise’ of Cas8f. PAM recognition also initiates unwinding of target DNA. Second, the target strand of target DNA hybridizes with the crRNA to form the R-loop structure, whose formation is accompanied by dramatic conformational changes in the Csy complex involving a rotation in Cas8f^HB^ (21). Third, the Cas2/3 nuclease is recruited to Cas8f^HB^ to cleave the target DNA (21).

**Figure 5.**
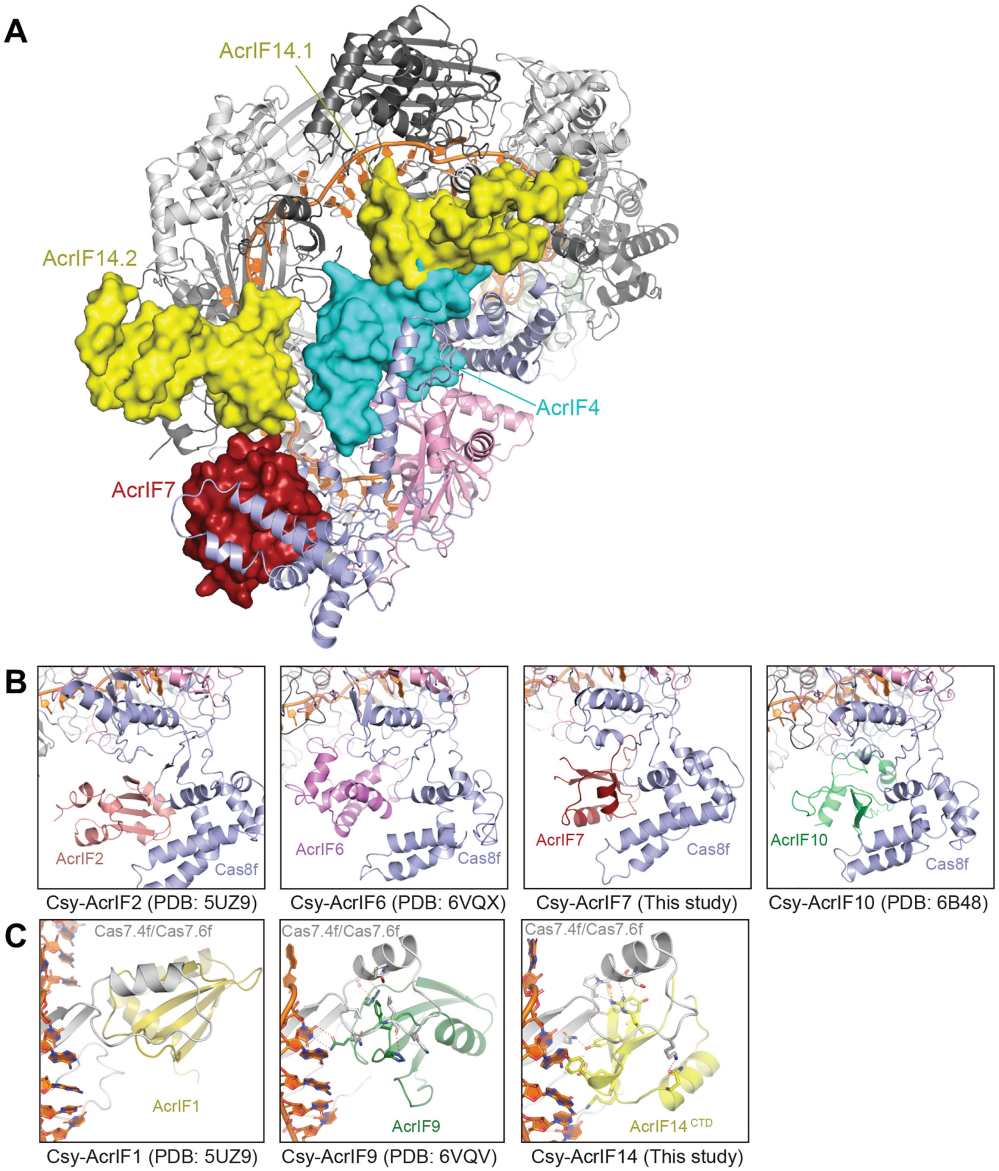
AcrIF4, AcrIF7 and AcrIF14 bind different sites of the Csy complex for its inhibition. (**A**) Binding sites of AcrIF4, AcrIF7 and AcrIF14 on the Csy complex. AcrIF4, AcrIF7 and AcrIF14 are shown in surface representation, whereas the Csy complex is in cartoon representation. (**B**) Side-by-side comparison of AcrIF proteins that bind to the DNA vise of Cas8f, including AcrIF2, AcrIF6, AcrIF7 and AcrIF10. (**C**) Side-by-side comparison of AcrIF proteins that bind to the thumb domains of Cas7.4f and Cas7.6f, including AcrIF1, AcrIF9 and AcrIF14.

Through structural determination, we showed that AcrIF7, AcrIF14 and AcrIF4 bind to different sites on the Csy complex to inhibit these three steps towards substrate degradation (Figure 5A). First, AcrIF7 binds to the DNA vise in the N-terminal domain of Cas8f and mimics the PAM duplex of target DNA. Such a mechanism was also utilized by AcrIF2 (18–20), AcrIF6 (23), and AcrIF10 (19), albeit there are no sequence or structural homology between these AcrIF proteins. (Figure 5B). Second, AcrIF14 binds to the thumb domains of Cas7.4f and Cas7.6f as well as the crRNA, blocking the hybridization between target DNA and the crRNA. Similar mechanisms were also seen in AcrIF1 (18–20) and AcrIF9 (22,23) (Figure 5C). Third, AcrIF4 primarily binds to the Cas8f^HB^, a binding site that was not reported in other AcrIF proteins. As a dramatic rotation in Cas8f^HB^ upon target DNA binding is required for recruitment of the Cas2/3 nuclease for target DNA cleavage (21), our structure indicates that AcrIF4 may preclude the conformational changes required for activation of the Csy complex. AcrIF4 has been characterized as a relatively weak inhibitor of the type I-F CRISPR–Cas system (41). This might be because the AcrIF4 binding site on the Csy complex is less well exposed. Our structural analysis of the Csy-AcrIF4 complex combined with EMSA data suggest that AcrIF4 does not affect dsDNA binding *in vitro*. However, we cannot rule out the possibility that AcrIF4 affects DNA binding *in vivo* as proposed before using a pyocyanin production assay (42).

Similar mechanisms of action from different AcrIF proteins indicate convergent evolution. The convergent evolution of Acr inhibitors is intriguing but not surprising. Phages contain proteins that are structurally and functionally similar with low sequence identity and similarity. Such shared structure and function is exemplified by the HK97-fold found in viral capsids, where proteins belonging to this family often only share 10–15% identity but great structural similarity (43,44). Thus, common mechanisms and features among AcrIF proteins targeting specific parts of the Csy complex further represent the convergent evolution common to MGEs.

Among the 14 AcrIF proteins, at least nine (AcrIF1, AcrIF2, AcrIF4, AcrIF6, AcrIF7, AcrIF8, AcrIF9, AcrIF10 and AcrIF14) stably bind to the Csy complex to inhibit recruitment of either target DNA or nuclease, suggesting direct association with the Csy complex is a major means for inhibition among AcrIF proteins. Other AcrIF proteins may adopt different inhibition mechanisms. For example, AcrIF3 targets the Cas2/3 nuclease and prevents its interaction with the Csy complex (21). Another attractive mechanism would be through enzymatic modification for inhibition, similar to the method of action of AcrVA1 (38,45) and AcrVA5 (46). Therefore, it will be interesting to test whether some AcrIF proteins work as enzymes. As an example, it was recently shown that AcrIF11 inactivates Csy by specifically ADP-ribosylating a key residue in the PAM-recognition loop, thereby inhibiting DNA binding (47). The discovery of an additional 10 AcrIF inhibitors (AcrIF15–24) (17) presents another interesting case as these newly discovered inhibitors may fall within one of the previously described methods of inhibition or lead to the discovery of new mechanisms against type I-F CRISPR–Cas systems.

## DATA AVAILABILITY

Cryo-EM reconstructions of Csy-AcrIF4, Csy-AcrIF7 and Csy-AcrIF14 complexes have been deposited in the Electron Microscopy Data Bank under the accession numbers EMD-22582, EMD-22583 and EMD-22585, respectively. Coordinates for atomic models of Csy-AcrIF4, Csy-AcrIF7 and Csy-AcrIF14 complexes have been deposited in the Protein Data Bank under the accession numbers 7JZW, 7JZX and 7JZZ, respectively.

## Supplementary Material

gkaa1199_Supplemental_Files
