# Supplementary material for: Structural basis for inhibition of the type I-F CRISPR–Cas surveillance complex by AcrIF4, AcrIF7 and AcrIF14
Source: Nucleic Acids Res. 2020 Dec 17;49(1):584–94. doi: 10.1093/nar/gkaa1199 (PMC7797054; doi:10.1093/nar/gkaa1199)
Supplement: gkaa1199_Supplemental_Files [file gkaa1199_supplemental_files.zip › Supplementary_Materials1120b.pdf]

Supplementary Materials

SUPPLEMENTARY FIGURES

Figure S1

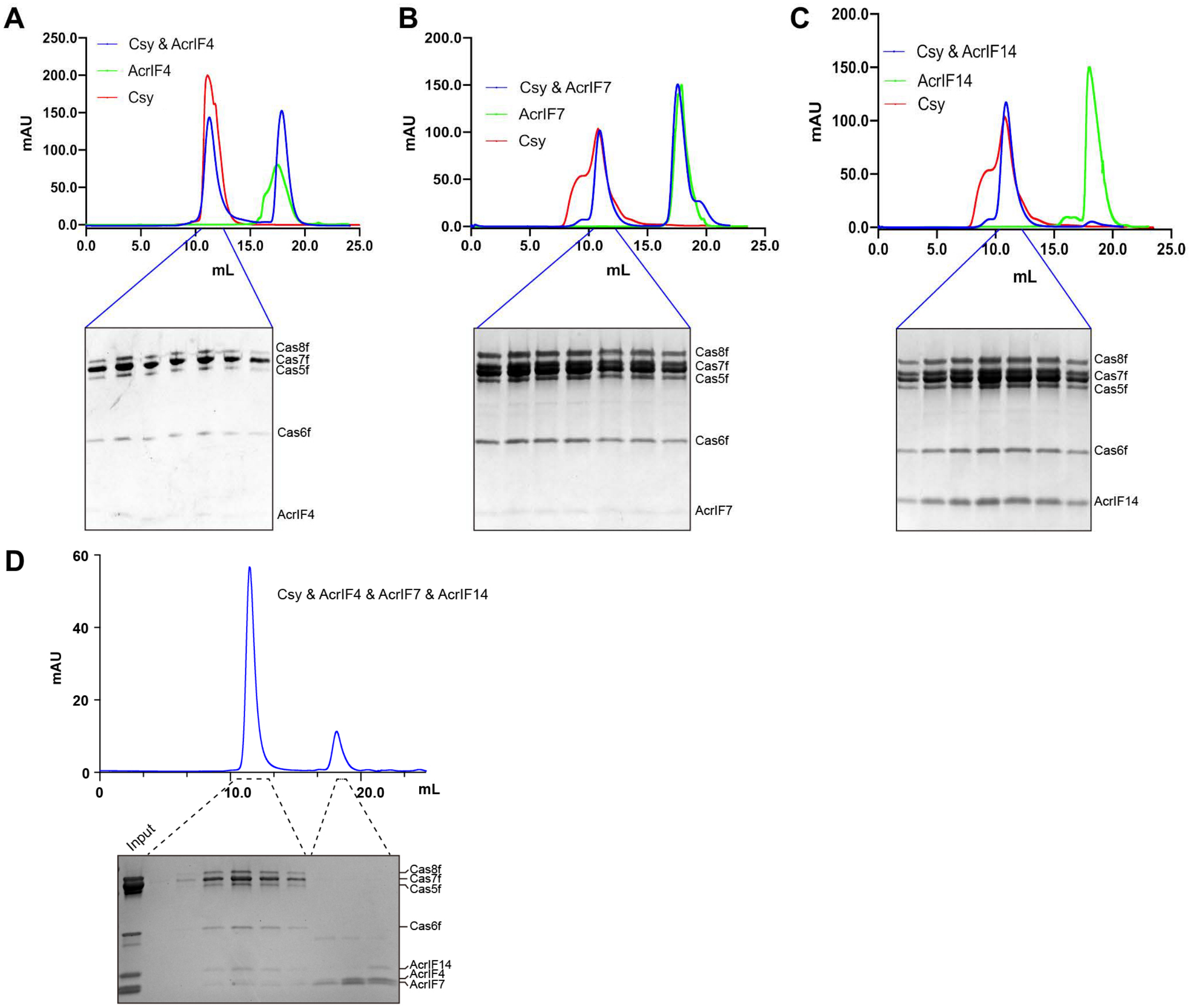

Figure S1. Binding assays between AcrIF proteins and the Csy complex. (A) Binding assay between AcrIF4 and the Csy complex. Upper: Size exclusion chromatography (SEC) profile. UV absorbance curves at 280 nm for AcrIF4 only (green line), the Csy complex only (red line), and incubated Csy complex and AcrIF4 (blue line) are shown. Lower: SDS-PAGE analysis of the elution fractions from SEC as indicated. **(B)** Binding assay between AcrIF7 and the Csy complex. **(C)** Binding assay between AcrIF14 and the Csy complex. **(D)** Binding assay between the Csy complex and all three AcrF proteins (AcrIF4, AcrIF7, and AcrIF14).

Figure S2

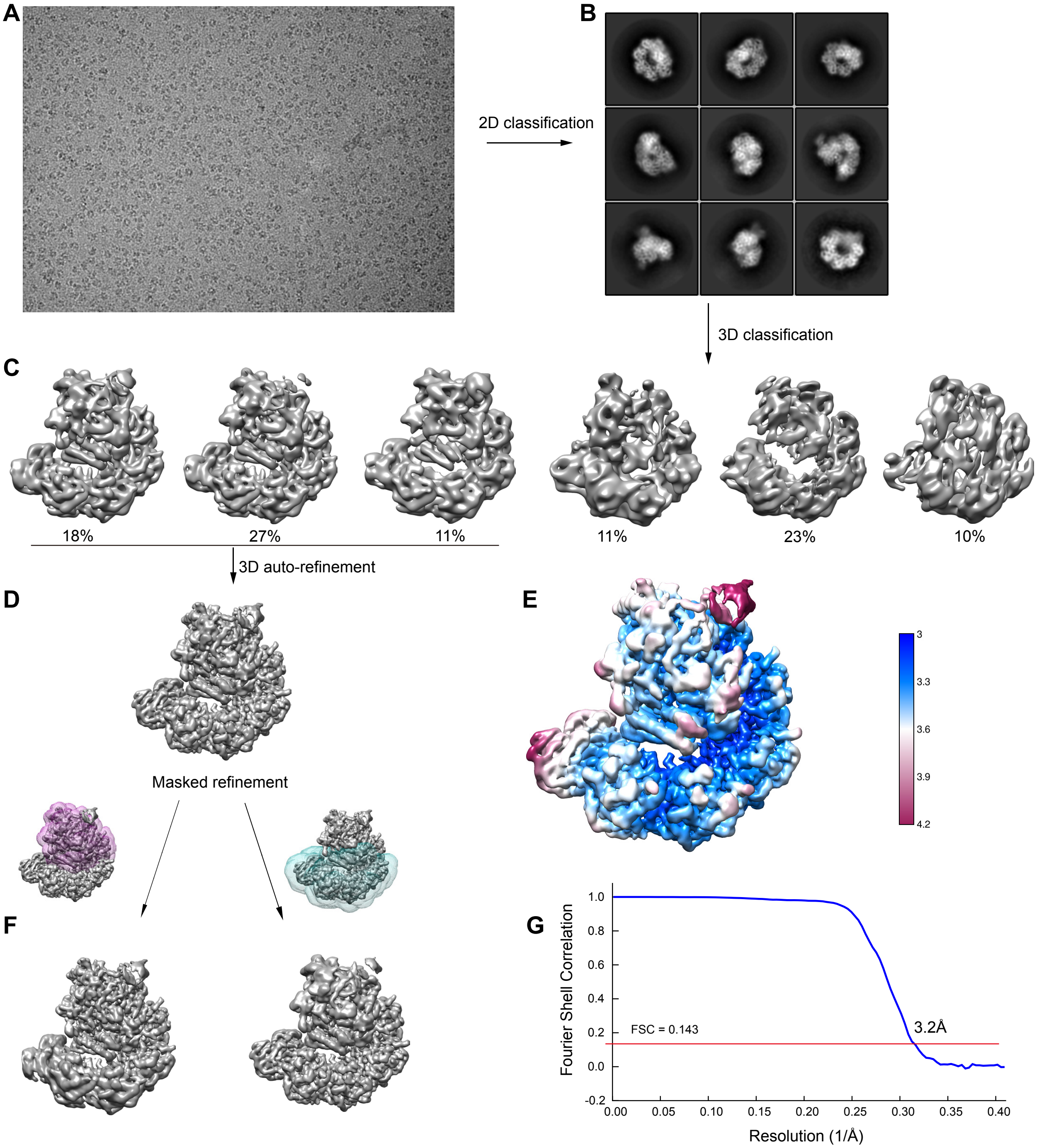

Figure S2. Cryo-EM of the Csy-AcrIF4 complex (A) A representative raw cryo-EM micrograph of the Csy-AcrIF4 complex. **(B)** Representative 2D class averages. **(C)** Major classes from 3D classification. **(D)** 3D refinement for particles from 3D classification as indicated. **(E)** Local resolution map for the reconstruction in **D**. **(F)** Two focused refinements with a soft mask either in the head or tail of the Csy complex to improve local resolutions. **(G)** Plot of the global half-map FSC indicates an average resolution of 3.2 Å.

Figure S3

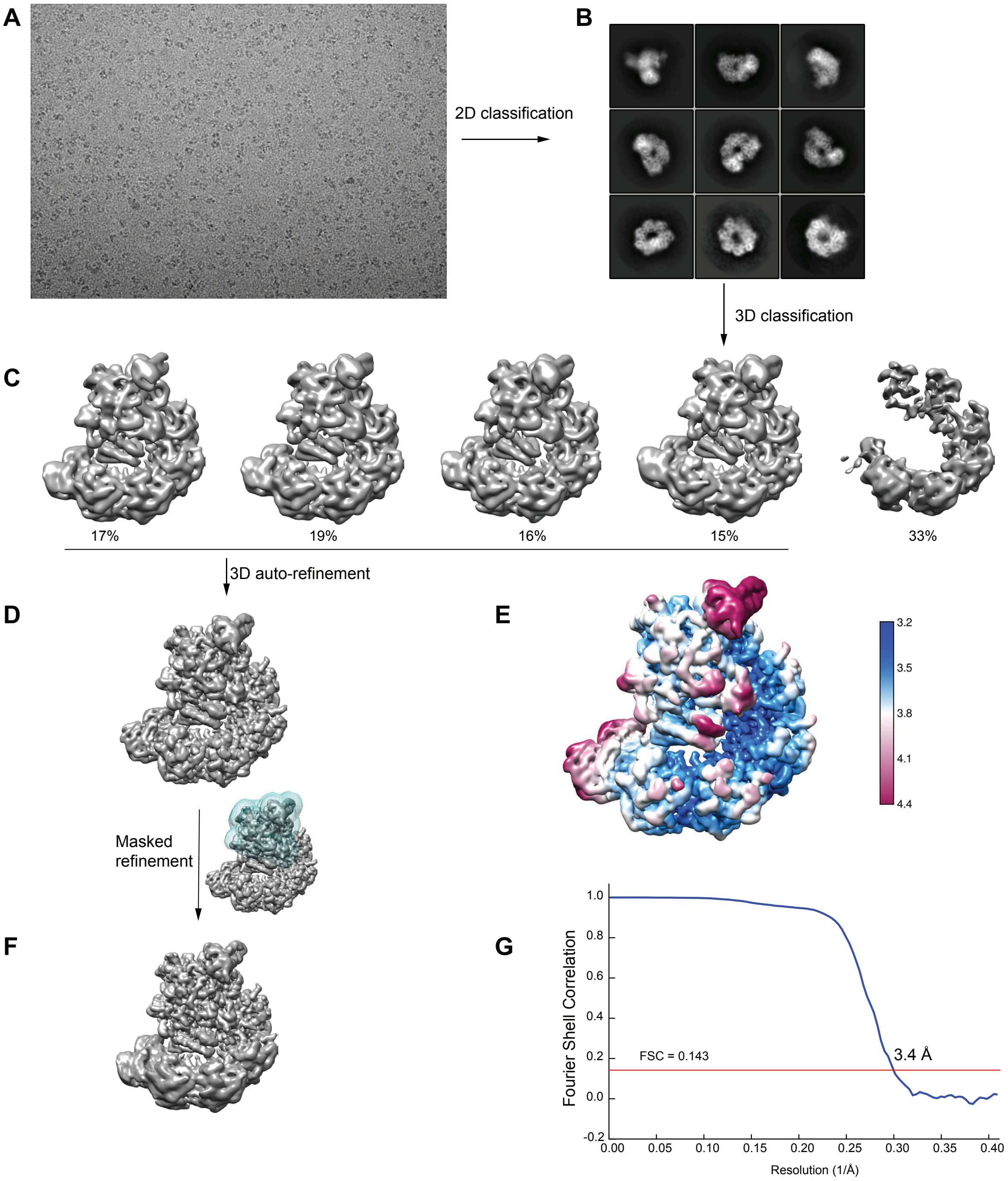

Figure S3. Cryo-EM of the Csy-AcrIF7 complex (A) A representative raw cryo-EM micrograph of the Csy-AcrIF7 complex. **(B)** Representative 2D class averages. **(C)** Major classes from 3D classification. **(D)** 3D refinement for particles from 3D classification as indicated. **(E)** Local resolution map for the reconstruction in **D**. **(F)** Focused refinement with a soft mask in the tail of Csy complex, where AcrIF7 is bound, to improve local resolutions. **(G)** Plot of the global half-map FSC indicates an average resolution of 3.4 Å.

Figure S4

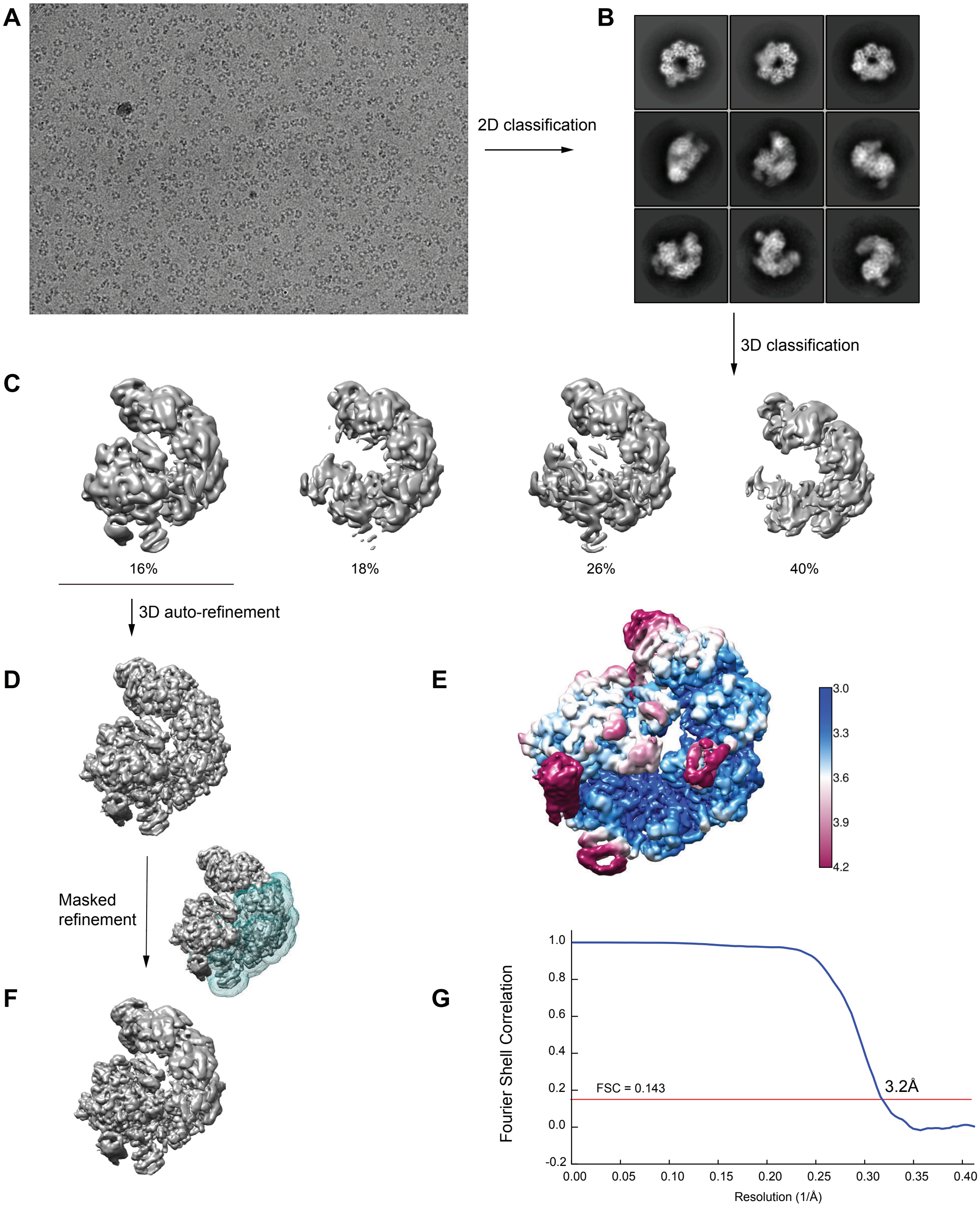

Figure S4. Cryo-EM of the Csy-AcrIF14 complex (A) A representative raw cryo-EM micrograph of the Csy-AcrIF14 complex. **(B)** Representative 2D class averages. **(C)** Major classes from 3D classification. **(D)** 3D refinement for particles from 3D classification as indicated. **(E)** Local resolution map for the reconstruction in **D**. **(F)** Focused refinement with a soft mask around AcrIF14 to improve local resolutions. **(G)** Plot of the global half-map FSC indicates an average resolution of 3.2 Å.

Figure S5

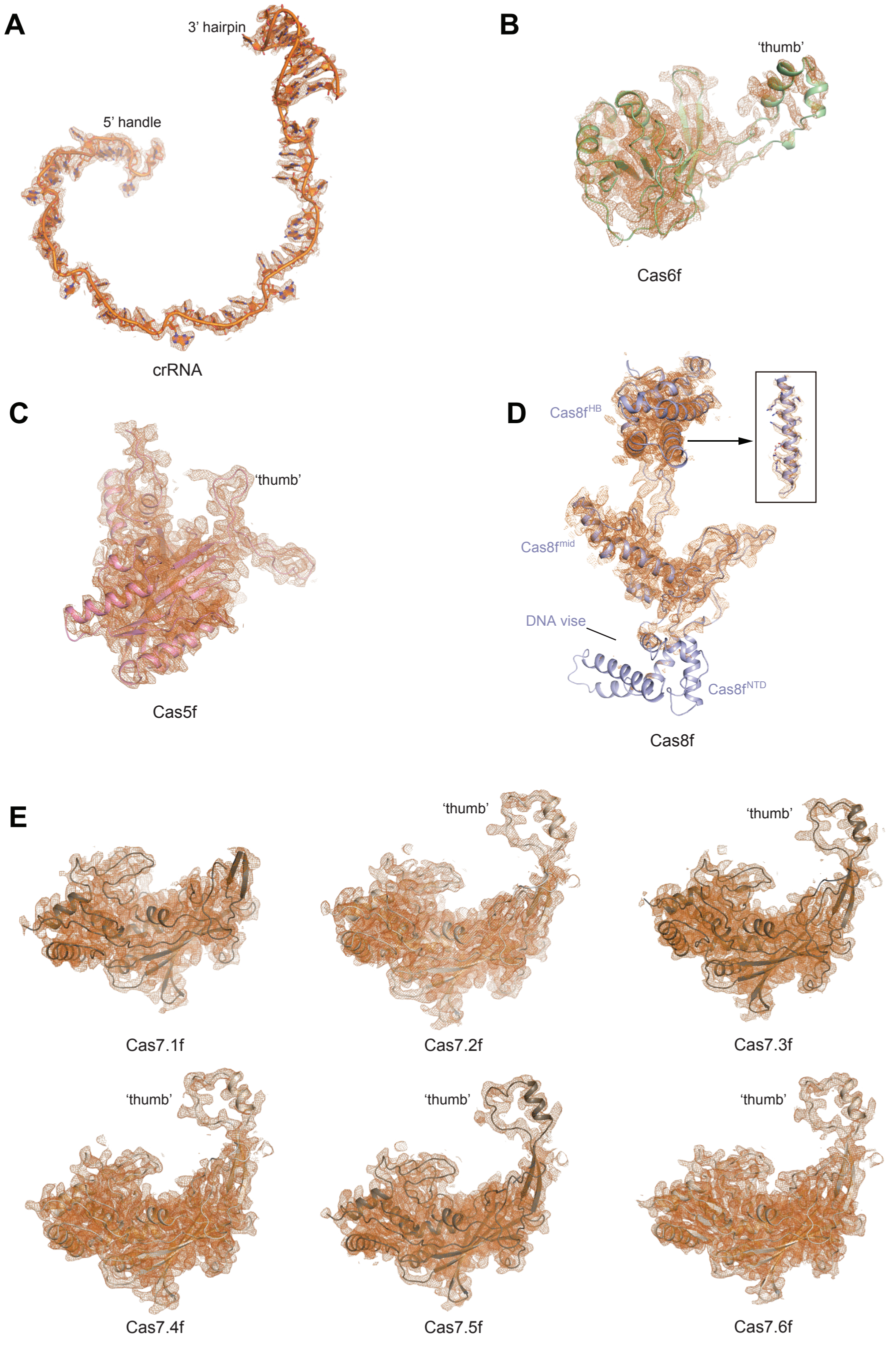

Figure S5. Structure of crRNA, Cas5f, Cas6f, Cas7f, and Cas8f in the Csy-AcrIF4 complex. **(A)** Structure of the crRNA. **(B)** Structure of Cas6f. **(C)** Structure of Cas5f. **(D)** Structure of Cas8f. Details of a helix in Cas8f^{HB} is shown on the right. **(E)** Structure of Cas7.1f-Cas7.6f. The corresponding cryo-EM density maps are shown in mesh.

Figure S6

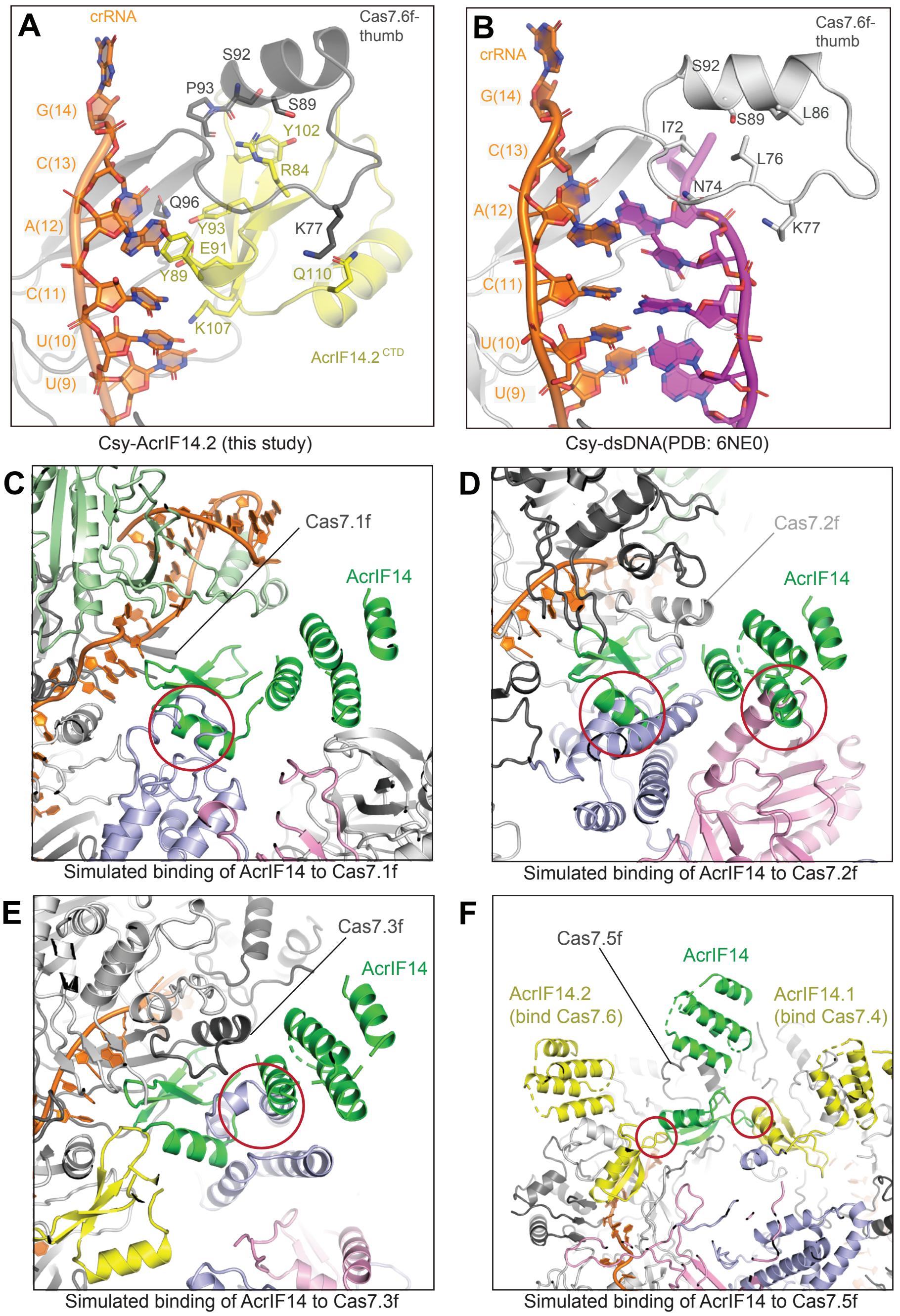

Figure S6. Interactions between AcrIF14.2 and Cas7.6f. (A) Detailed interactions between AcrIF14.2 and Cas7.6f, shown as the same view as in **Fig.4C**. **(B)** Detailed interactions between target DNA and Cas7.6f, shown in the same view as in **A**. **(C-F)** Simulated binding of AcrIF14 to Cas7.1f, Cas7.2f, Cas7.3f, and Cas7.6f. Red circles indicate steric clashes between simulated AcrIF14 (green) and the Csy complex or AcrIF14 bound to Cas7.4f and Cas7.6f (yellow).

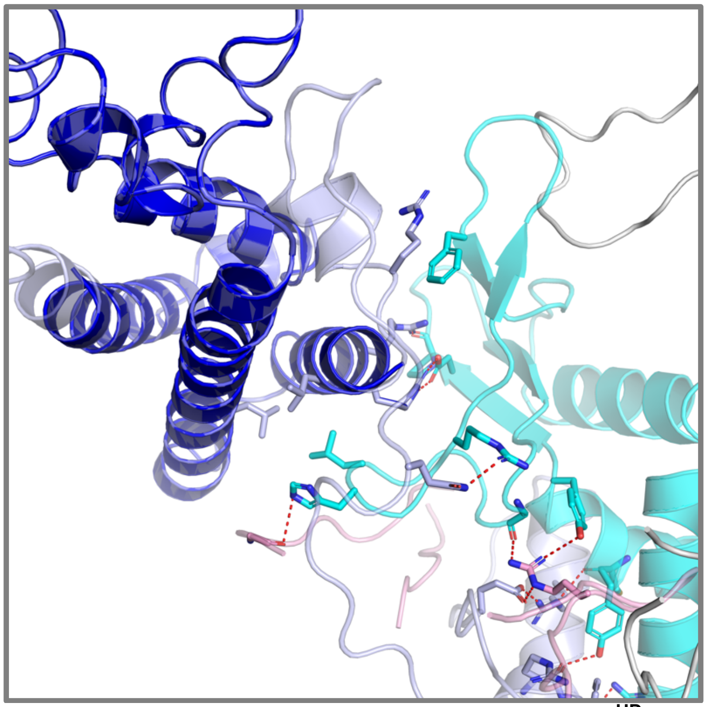

Movie S1. Conformational changes of Cas8f^{HD} upon substrate DNA binding. This movie starts with a view showing interactions between AcrF4 (in cyan) and Cas8f^{HD} (in light blue) as shown in Figure 2F. Conformational changes in Cas8f^{HD} upon substrate DNA binding (in blue; PDB: 6NE0) is then displayed.
